# Broccoli extract increases drug-mediated cytotoxicity towards cancer stem cells of head and neck squamous cell carcinoma

**DOI:** 10.1038/s41416-020-1025-1

**Published:** 2020-08-10

**Authors:** Osama A. Elkashty, Simon D. Tran

**Affiliations:** 1 McGill Craniofacial Tissue Engineering and Stem Cells Laboratory, Faculty of Dentistry, McGill University, Montreal, QC Canada; 2 Oral Pathology Department, Faculty of Dentistry, Mansoura University, Mansoura, Egypt

**Keywords:** Cancer stem cells, Oral cancer, Cancer therapy

## Abstract

**Background:**

Head and neck squamous cell carcinomas (HNSCC) are malignant neoplasms with poor prognosis. Treatment-resistant cancer stem cell (CSC) is one reason for treatment failure. Considerable attention has been focused on sulforaphane (SF), a phytochemical from broccoli possessing anticancer properties. We investigated whether SF could enhance the chemotherapeutic effects of cisplatin (CIS) and 5-fluorouracil (5-FU) against HNSCC–CSCs, and its mechanisms of action.

**Methods:**

CD44^+^/CD271^+^ FACS-isolated CSCs from SCC12 and SCC38 human cell lines were treated with SF alone or combined with CIS or 5-FU. Cell viability, colony- and sphere-forming ability, apoptosis, CSC-related gene and protein expression and in vivo tumour growth were assessed. Safety of SF was tested on non-cancerous stem cells and in vivo.

**Results:**

SF reduced HNSCC–CSC viability in a time- and dose-dependent manner. Combining SF increased the cytotoxicity of CIS twofold and 5-FU tenfold, with no effects on non-cancerous stem cell viability and functions. SF-combined treatments inhibited CSC colony and sphere formation, and tumour progression in vivo. Potential mechanisms of action included the stimulation of caspase-dependent apoptotic pathway, inhibition of SHH pathway and decreased expression of SOX2 and OCT4.

**Conclusions:**

Combining SF allowed lower doses of CIS or 5-FU while enhancing these drug cytotoxicities against HNSCC–CSCs, with minimal effects on healthy cells.

## Background

Head and neck squamous cell carcinoma (HNSCC) is the seventh most common malignancy worldwide, represents ~6% of all cancer cases and is accounted for 580,000 new cases and over 380,000 deaths annually.^1–3^ The current standard treatment for HNSCC is by multimodal approaches consisting of surgery, radiotherapy and/or chemotherapy.^4^ Despite advances in diagnostic tools and treatment modalities, the 5-year survival rate of HNSCC remains at 50%.^5,6^ Cancer stem cells (CSCs), also known as tumour-initiating cells, are a distinct cell subpopulation within the tumour.^7^ When compared with the remaining tumour cells, CSCs are often more resistant to chemoradiotherapy and more tumorigenic.^8^ Therefore, it is of great importance to develop strategies for targeting CSCs in order to improve HNSCC treatment outcomes.

Sulforaphane (SF), a phytochemical that exists in a large amount in cruciferous plants, has shown promising anti-inflammatory, anti-oxidant and antitumour effects.^9–12^ Recent studies have proposed that SF exerts its antitumour effects through inhibiting both cell proliferation and cell- cycle mechanisms, promoting apoptosis and protecting the precancerous cells from methylation.^12,13^ However, its effect on CSCs in HNSCC, either alone or in combination with conventional chemotherapy, remains poorly understood.^14^ Therefore, our present study was designed to investigate whether SF could be a potent agent to enhance the chemotherapy efficacy of cisplatin (CIS) and 5-fluorouracil (5-FU) on HNSCC stem cells, and to determine its mechanisms of action.

## Methods

### Cell culture

UM-SCC12 (laryngeal SCC, RRID: CVCL_7717) and UM-SCC38 (tonsillar SCC, RRID: CVCL_7749) human cell lines were purchased from University of Michigan in 2015 and used as models for HNSCC.^13^ These cell lines have been authenticated using STR analysis in 2019 at Genome Quebec. They were cultured in Dulbecco’s modified Eagle medium (DMEM, Thermo Fisher, Waltham, Massachusetts, United States) supplemented with 1% non-essential amino acids. Periodontal ligament stem cells (PDLSCs) and dental pulp stem cells (DPSCs) were isolated from extracted teeth and cultured in minimum essential medium (MEM, Thermo Fisher).^15^ Both culture media were supplemented with 10% foetal bovine serum and 1% antibiotic–antimycotic (Thermo Fisher). All cell types were mycoplasma-free and were incubated in a humidified incubator at 37 °C with 5% CO_2_.

### Cytotoxic agents

Sulforaphane (Cayman Chemical, Ann Arbor, Michigan, United States) was purchased as a solution in ethanol with purity ≥ 98% and stored at −20 °C. Cisplatin (Cayman Chemical) was prepared in phosphate-buffered saline (PBS) to a 0.3 mg/ml stock and was kept protected from light at 4 °C. 5-Fluorouracil (Sigma Aldrich, St. Louis, Missouri, United States) was prepared in dimethyl sulfoxide (DMSO) to 50 mg/ml stock. The final concentrations of the solvents, either PBS or DMSO, in the working solution medium were 0.1% or less.

### Fluorescence‑activated cell sorting (FACS)

Flow cytometry and fluorescence‑activated cell sorting was performed as previously described.^16^ Briefly, Alexa Fluor^®^ 700 Mouse Anti-Human CD44 (Clone G44-26) and PerCP-Cy™ 5.5 Mouse Anti-Human CD271 (Clone C40-1457) monoclonal antibodies were obtained from BD Pharmingen. Tumour cells were harvested using Accutase™ (BD Bioscience, San Jose, Canada) and resuspended with a final concentration of 1 × 10^6^ cells/100 µl for the staining procedures. FACS of CD44^+^/CD271^+^ cells was performed using a BD FACSARIA III cells sorter (BD Bioscience). UltraComp eBeads™ Compensation Beads (Thermo Fisher) were used as control.

### MTT assay for cell viability

As described previously,^13^ 1500 cells per well were seeded in 96-well plates, and they were treated with different concentrations of SF and/or chemotherapeutic agents for 72 h. The medium was then removed, and 10% solution of 5 mg/ml MTT in medium (Sigma Aldrich) was added and incubated at 37 °C for 2 h. Formazan was dissolved by adding DMSO to each well after MTT removal. The optical density was measured at 562/540 nm in EL800 Microplate Reader (BIO-TEK Instruments, Winooski, Vermont, United States). For analysing the effect of SF over time, cells were treated with 3.5 µM SF, and the same steps were followed daily for 4 consecutive days.

### Colony-forming assay

CD44^+^/CD271^+^ cells were seeded at 1 × 10^5^ cells/well in 6-well tissue culture plates. The cells were treated with SF and/or chemotherapeutic agents for 72 h. Then, cells were detached, plated at a density of 400 single living cells/well in 6-well tissue culture plates and incubated for 10 days while the medium was being changed every 3 days. The cell colonies were fixed and stained with 1% crystal violet, 50% methanol in DDH_2_O for 1 h. The number of colonies with >50 cells were counted under an inverted microscope.

### Sphere-forming assay

In total, 5000 CD44^+^/CD271^+^ cells/500 µl per well were seeded in 24 ultra-low-attachment multiple-well plate (Millipore Sigma, Burlington, Massachusetts, United States) in DMEM-F-12 medium (Thermo Fisher) reconstituted with 20 ng/ml of epidermal growth factor, 20 ng/ml of basic fibroblast growth factor, 0.5% N_2_ supplement (STEMCELL Technologies, Vancouver, Canada), 1% B27 supplement and 2% antibiotic–antimycotic (Thermo Fisher). After 24 h, SF and/or the two chemotherapeutic agents were added. The medium with drugs was added every 2–3 days to measure the long-term effect on cells. Photographs of groups were captured at 14 days, using a phase-contrast microscope.

For serial passage, single cells were obtained from Accutase-treated spheroids. Then, the same steps were followed as described above. Spheres were then collected by centrifugation and dissociated by Accutase to single cells to obtain a cell count.

### Annexin V apoptosis detection

Post-treatment apoptosis was measured by using the PE-Annexin V Apoptosis Detection Kit (BD Bioscience). Briefly, 1.5 × 10^5^ CSCs from SCC12 cell line were seeded per well, in a 6-well plate for 24 h, and were then treated with SF and/or the chemotherapeutic agents for 72 h. Cells were detached using Accutase (Biolegend, San Diego, California, United States); then, all procedures followed the manufacturer’s protocol. Cells were analysed by flow cytometry using LSR Fortessa (BD Biosciences). Data analysis was performed using FlowJo vX (FlowJo LCC).

### Real-time qRT-PCR

Gene expression levels in CD44^+^/CD271^+^ cells from the SCC12 cell line after exposure to SF and/or chemotherapeutic agents for 3 days were measured as previously described.^16^ Glyceraldehyde-3-phosphate dehydrogenase (GAPDH) was used as the endogenous expression standard. The appendix lists the gene-specific sequence of the primers. Gene expression was calculated based on ΔΔCt method. The *n*-fold difference in mRNA expression was determined according to the method of 2^−ΔΔCT^.

### Western blot assay

CD44^+^/CD271^+^ cells from the SCC12 cell line were exposed to SF and/or chemotherapeutic agents for 3 days, then harvested using trypsin. A lysis buffer that consisted of 10 mM Tris-HCl, pH 7.2, 150 mM NaCl, 5 mM ethylenediaminetetraacetic acid (EDTA), 1% Triton X-100, 0.1% sodium dodecyl sulfate (SDS) and 1% Na-deoxycholate was used to lyse the cells. After centrifugation at 15,000 × *g* for 20 min, supernatants were recovered, and the protein content was quantified by the Pierce™ BCA Protein Assay Kit (Thermo Fisher). Protein samples (20–60 μg) were size-separated by electrophoresis on sodium dodecyl sulfate-polyacrylamide gels under reducing conditions. Separated proteins were electroblotted onto nitrocellulose membranes. Non-specific immunoreactions in the blot were blocked by 5% skim milk and incubated with one of the following primary antibodies: anti-human BMI1, anti-BCL2, anti-active Caspase 3 (Cell Signaling, Danvers, Massachusetts, United States), anti-SOX2, Anti-OCT4 and anti-β actin (Abcam, Cambridge, United Kingdom) overnight at 4 °C. Horseradish peroxidase (HRP)-conjugated anti-goat or -rabbit secondary antibody was then used. Antibody-bound proteins were detected by the spray on ECL (Zmtech Scientifique, Montreal, Canada) and ChemiDoc™ Touch Imaging System (Bio-Rad, Hercules, California, United States).

### Osteogenic differentiation

DPSCs and PDLSCs were treated with 3.5 µM SF for 3 days; then, the cells were collected and seeded in 6-well plates, 2 × 10^5^ cells/well, and allowed to grow to 70% confluency in normal medium. Thereafter, the growth media were replaced with the osteogenic medium containing α-MEM supplemented with 1% antibiotic/antimycotic, 20% FBS, 2 mM glutamine, 10^−8^ M dexamethasone sodium phosphate, 55 µM 2-mercaptoethanol, 0.1 mM l-ascorbic acid and 2 mM β-glycerophosphate. Control cells were cultured in the normal growth medium. Both media were changed every 3 days. All cultures were allowed to grow for 21 days, then fixed and stained with Alizarin Red (Sigma). Photographs of all groups were captured using a phase-contrast microscope at ×5 magnification. Osteogenic quantification was done by unbinding the Alizarin Red stain using 10% (v/v) acetic acid followed by reading the absorbance at a wavelength of 405 using microplate reader.

### Chondrogenic differentiation

DPSCs and PDLSCs were treated with 3.5 µM SF for 3 days; then, the cells were collected as 5 × 10^5^ cells in 15-ml polypropylene tubes. Cells were centrifuged, and the media were replaced with the StemXVivo Chondrogenic Base Media supplemented with StemXVivo Chondrogenic Supplement (R&D Systems, Minneapolis, Minnesota, United States) and 1% antibiotic/antimycotic. Control cells were cultured in the normal growth medium. Every 3 days, half of the medium was replaced by a new medium. All cultures were grown for 21 days; then, the pellets were collected and frozen by OCT compound (Thermo Fisher), cryosectioned and stained by Collagen Type II immunofluorescent staining. Photographs were captured using a phase-contrast microscope at ×20 magnification. Chondrogenic quantification was done using ImageJ software (NIH).

### In vivo assay and tumour xenografts

For the in vivo experiments, we used SF and CIS only, without 5-FU to decrease the number of mice to be sacrificed. This animal research study was approved by the University Animal Care Committee at McGill University (Protocol #5330, www.animalcare.mcgill.ca) and conformed to ARRIVE (animal research: reporting of in vivo experiments) guidelines. The animals used in this study were 23 NU/NU nude (Crl:NU-Foxn1^nu^) mice (*n* = 5 in each group and *n* = 3 in the sham-control group) (Charles River, Wilmington, Massachusetts, United States). All the mice were kept in clean conditions with soft food and water in the animal resource centre at McGill University. Six- to ten-week-old male mice were injected with 1 × 10^4^ CD44^+^/CD271^+^ SCC12 cells (suspended in 30 μl of normal saline) on the lateral side of the tongue using a 1-ml tuberculin syringe with a 30-gauge hypodermic needle, under general anaesthesia with isoflurane (Isoba Vet^TM^). After 1 week, mice-bearing tumours were randomly divided into groups, and different (drug) treatments were administered. Mice were injected intraperitoneally (I.P) with the vehicle control (normal saline), SF (4 mg/kg), CIS (3 mg/kg) or a combination of SF and CIS every 3 days for a total of 6 doses.^17^ Mice were examined weekly to measure their body weight and the tumour size bidirectionally using a calibre, under isoflurane gas anaesthesia. Tumour size was calculated using the following formula: volume = (width)^2^  * length/2. Animals were sacrificed after 49 days with CO_2_ inhalation, and the tongues, livers and kidneys were collected. Tumour formation in the tongues, and liver or kidney necrosis was assessed using H&E-stained sections.

### Statistical analysis

Data were presented as the means ± standard deviations (SD) of three independent experiments conducted in triplicate with comparable results. One-way analysis of variance (ANOVA) followed by post hoc Tukey’s test was used to assess significant differences between three groups or more, while Student’s *t* test (Unpaired) was used to compare two groups. *p* Values < 0.05 were considered statistically significant. GraphPad Prism 6 software was used for the statistical analysis (GraphPad Software, San Diego, Canada).

## Results

### Effects of sulforaphane on the viability and proliferation in HNSCC–CSCs

**Fig. 1:**
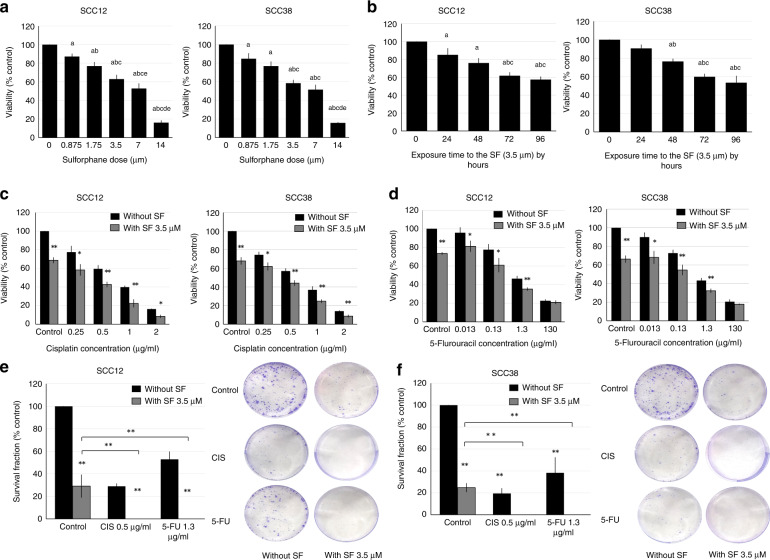
Effects of sulforaphane on the viability and proliferation in HNSCC–CSCs. **a** HNSCC–CSCs were treated with 0, 0.875 μM, 1.75 μM, 3.5 μM, 7 μM or 14 μM of SF for 72 h. Data are presented as means ± SD for *N* = 3 (“a” means a *P* value < 0.05 relative to 0 μM, “b” to 0.875 μM, “c” to 1.75 μM, “d” to 3.5 μM and “e” to 7 μM). **b** HNSCC–CSCs were treated with 3.5 μM of SF for the indicated times (“a” significance relative to 0 h, “b” significance relative to 24 h and “c” significance relative to 48 h. *P* < 0.05). HNSCC–CSCs were treated with 3.5 μM of SF with or without 0.1, 0.5, 1 or 2 μg/ml of CIS (**c**), or 0.013, 0.13, 1.3 or 130 μg/ml of 5-FU (**d**) for 72 h. Data are presented as means ± SD for *N* = 3 (*P < 0.05 and **P < 0.01 relative to treatment in the absence of SF). **e** HNSCC–CSCs were pre-treated with SF with or without CIS or 5-FU for 72 h before being seeded in 6-well plates for 10 days. Fixed and stained colonies containing >50 cells were counted under an inverted light microscope. Data were presented as means ± SD for *N* = 3 (**P < 0.01). Photographs of the fixed and stained colonies are presented in (**f**).

FACS-isolated CSCs were exposed to different SF concentrations. SF treatment decreased the viability of HNSCC–CSCs in a dose-dependent manner (Fig. 1a). The half-maximal inhibitory concentration (IC50) of SF on CSCs was 5.54 μM for SCC12 and 5.13 μM for SCC38. The inhibitory effects of SF on cellular viability increased over time, as shown by testing CSCs to 3.50 μM SF (Fig. 1b).

Adding 3.50 μM SF had a statistically significant increase in the inhibition of cell viability when compared with using either CIS (Fig. 1c) or 5-FU (Fig. 1d) as a single chemotherapy for all the tested drug concentrations. The effect was nearly doubled with CIS (Fig. 1c) and increased ten times with 5-FU (Fig. 1d), especially at the lower chemotherapeutic concentrations.

By using 3.50 μM of SF alone, the clonogenic ability of the CSCs was reduced to 29% ± 10.1 and 24% ± 3.9% in SCC12 and SCC38, respectively, when compared with the control, and this was comparable to using 0.5 μg/ml of CIS alone. CIS as a single treatment also reduced the clonogenic ability to 28% ± 2.4 and 19% ± 4.6%, while 5-FU reduced it to 52% ± 6.8 and 38% ± 14% for SCC12 and SCC38, respectively. Surprisingly, the combination of SF + CIS or SF + 5-FU completely prevented CSC colony formation (Fig. 1e, f).

### Effect of sulforaphane on self-renewal and apoptosis in HNSCC–CSCs

**Fig. 2:**
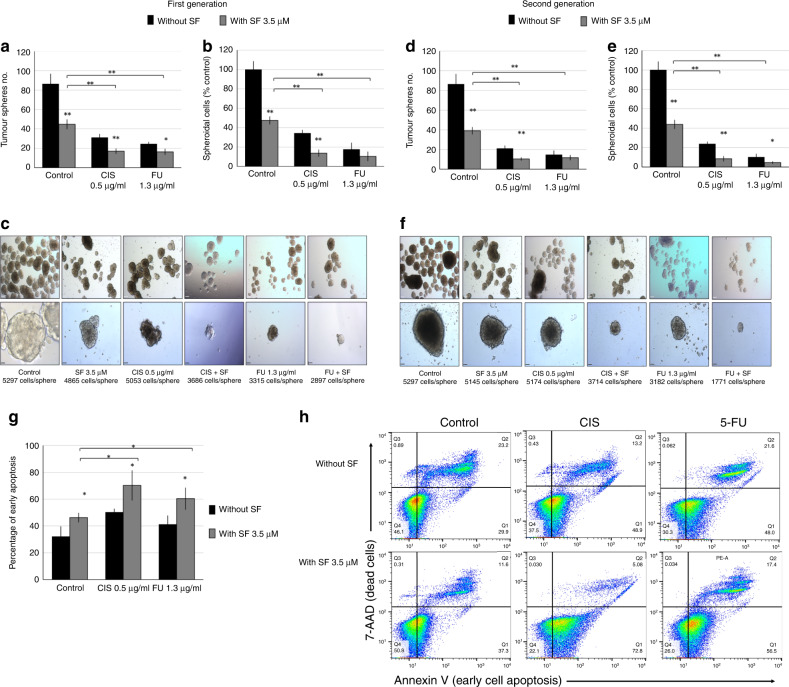
Effect of sulforaphane on the self-renewal ability and apoptosis induction of HNSCC–CSCs. HNSCC–CSCs were seeded at a clonal cell density (5000 cells/500 µl per well) in ultra-low-attachment plates for spheroid formation. Twenty-four hours later, cells were treated with SF and/or CIS, or 5-FU, and allowed to form spheres for 14 days. The spheres were counted (**a**), dissociated into single cells and counted using trypan blue staining (**b**). **c** Representative photographs of the first generation of spheres formed under ×5 (upper panel) and ×40 (lower panel) magnifications (scale bars of 90 µm and 10 µm, respectively). **d**–**f** Spheroids were dissociated into single cells, and an equal number of live cells were re-plated. Fourteen days later, the second generation of spheroids were formed. These second-generation spheroids were photographed and dissociated again for cell counting. Data are presented as means ± SD for *N* = 3 (*P < 0.05, **P < 0.01 relative to treatments in the absence of SF). **h** The percentage of early apoptotic cells was presented as means ± SD for *N* = 3 (*P < 0.05). **g** Flow cytometry graphs showing the gating strategy. The vertical line represents the cutline for Annexin V staining, and the horizontal line represents the cutline for 7-aminoactinomycin D (7-AAD) staining.

While single treatment with SF, CIS or 5-FU reduced spheroid formation, the combinations SF + CIS or SF + 5-FU inhibited spheroid formation most effectively (Fig. 2a, c). The effect was not only on the number of spheres, but also on the size of the formed spheres; the combination treatments produced smaller spheres with fewer cell numbers (Fig. 2b, d). Furthermore, the combinations SF + CIS or SF + 5-FU also inhibited the formation of secondary spheres (both their numbers and sizes, Fig. 2e, f).

SF treatment alone induced early apoptosis in 46% ± 3.4% of CSCs as compared with 32% ± 7.3% in the control group. CIS treatment induced early apoptosis to 50.3% ± 2.4% CSCs, while combining SF + CIS increased apoptosis to 70.2% ± 11.1%. Similarly, 5-FU as a stand-alone treatment induced apoptosis to 41.2% ± 6.4% CSCs, while the combined treatment SF + 5-FU increased apoptosis to 60.3% ± 8.1% (Fig. 2g, h). These results suggested that SF could reduce HNSCC–CSC numbers through the induction of apoptosis, in addition to inhibiting cell proliferation and self-renewal.

### Effect of sulforaphane on the genotyping of HNSCC–CSCs

**Fig. 3:**
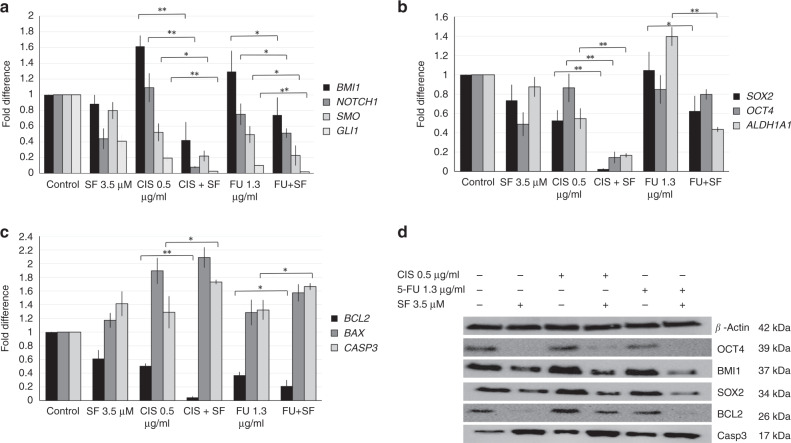
Effect of sulforaphane on the genotype of HNSCC–CSCs. HNSCC–CSCs were treated with 3.5 μM of SF with or without 0.5 μg/ml of CIS or 1.3 μg/ml of 5-FU for 72 h. The expression of (**a**) self-renewal-related genes (BMI1, SMO, GLI1 and NOTCH1), **b** stemness and drug resistance-related genes (SOX2, OCT4 and ALDH1A1) and **c** apoptosis-related genes (BAX, BCL2 and CASP3) was measured by qRT-PCR and normalised to GAPDH expression. Data represent means ± SD for *N* = 3 (*P < 0.05 and **P < 0.01 relative to treatments in the absence of SF). **d** Proteins were harvested from the treated cells, and expression of OCT4, BMI1, SOX2, BCL2 and activated Caspase 3 proteins was analysed by western blot. Expression of β-actin served as the loading control.

By combining SF to either CIS or 5-FU, there was a significant decrease in the expression levels of NOTCH1, SMO and GLI1 genes when compared with using CIS or 5-FU alone. This led to inhibiting their downstream gene BMI1 (Fig. 3a).

Combining SF + CIS or SF + 5-FU decreased SOX2 expression significantly when compared with either CIS or 5-FU alone. The decrease in OCT4 gene expression was significant only for the SF + CIS combination treatment. Drug resistance and stemness-related mRNA expression level of ALDH1A1 was analysed by qRT-PCR (Fig. 3b). SF combination with CIS or 5-FU significantly reduced ALDH1A1 mRNA expression level when compared with single CIS or 5-FU chemotherapy treatment (Fig. 3b).

Our results showed a significant decrease in BCL2 expression after combining SF + CIS or SF + 5-FU, and although there was an increase in the expression of BAX with the combined treatment, it was not significant. Caspase 3 expression was elevated with SF + CIS or SF + 5-FU when compared with using each chemotherapy alone (Fig. 3c). qRT-PCR results were confirmed by western blotting to detect the changes at the protein level and the activation of Caspase 3 by cleavage (Fig. 3d).

### Effect of sulforaphane on non-cancerous (healthy) stem cells

**Fig. 4:**
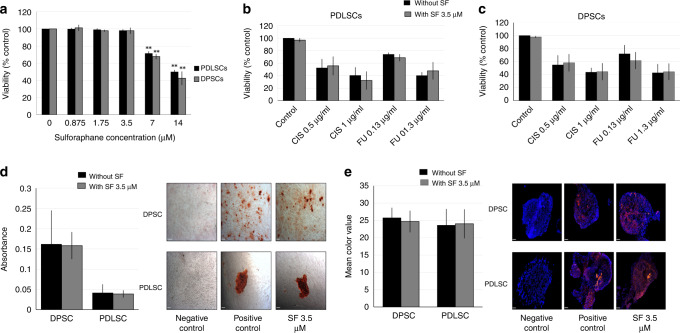
Effect of sulforaphane on non-cancerous human stem cells. **a** Periodontal ligament stem cells (PDLSCs) and dental pulp stem cells (DPSCs) were treated with 0, 0.875, 1.75, 3.5, 7 and 14 μM of SF for 72 h. Data are presented as means ± SD for *N* = 3 (**P < 0.01 compared with the control). **b** PDLSCs and **c** DPSCs were treated with 3.5 μM of SF in the presence or absence of 0.5 and 1 μg/ml of CIS, or 0.13 and 1.3 μg/ml of 5-FU for 72 h, respectively. Data are presented as means ± SD. **d** PDLSCs and DPSCs were treated with 3.5 µM SF for 72 h, induced to undergo osteogenic differentiation and then stained with Alizarin Red for identification and quantification of osteocytes. Photographs were taken at ×5 magnification, scale bar = 90 µM. **e** PDLSCs and DPSCs were treated with 3.5 µM SF for 72 h, induced to undergo chondrogenic differentiation and then cryosectioned and stained with Collagen Type II immunofluorescence staining for the identification and quantification of chondrocytes. Photographs at 20× magnification, scale bar = 47 µM.

The effects of SF alone or combined with CIS or 5-FU were tested on non-cancerous human stem cells (nCSCs), such as periodontal ligament stem cells (PDLSCs) and dental pulp stem cells (DPSCs). SF alone did not show any significant toxicity to nCSCs in concentrations less than 3.5 μM (Fig. 4a). There was no significant difference between using CIS or 5-FU as a single treatment versus the addition of SF (Fig. 4b, c).

There was no significant difference between SF-treated and -untreated PDLSCs and DPSCs in their abilities for osteogenic (Fig. 4d, e) or chondrogenic differentiation (Fig. 4f, g).

### Effect of the SF + CIS combination treatment in vivo

**Fig. 5:**
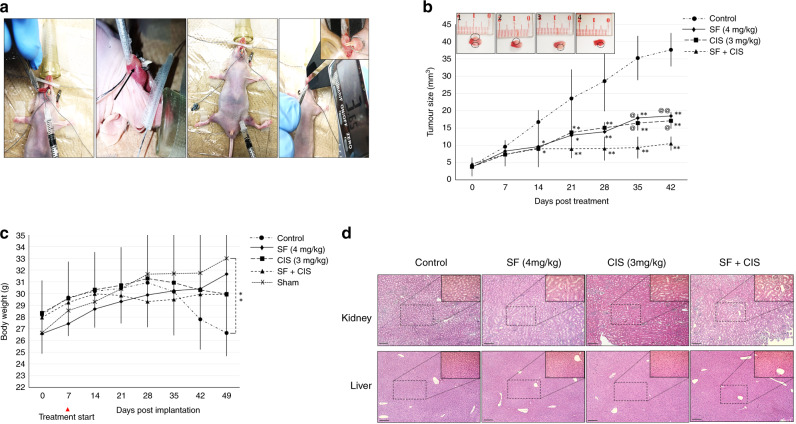
In vivo effect of combining SF + CIS treatment on cancer stem cells in a xenograft HNSCC mouse model. **a** Intra-oral tongue xenografts of HNSCC–CSCs in nude immunocompromised mice that were treated with IP injections of the vehicle control (normal saline), SF, CIS or SF + CIS (*n* = 5 mice per group). Intra-oral tumour size and mice body weights were monitored weekly. Black arrow indicates tumour formation after 1 week of tumour implantation with 1 × 10^4^ CD44^+^/CD271^+^ SCC12 cells. **b** Tumour volumes and **c** mice body weights were determined as described in the “Methods” section. Data represent means ± SD for *N* = 5 (*P < 0.05 and **P < 0.01 compared with the control, ^@^*P* < 0.05 and ^@^*P* < 0.01 compared with the combined treatment). **d** A representative H&E staining of histological sections of the kidneys (upper row) and livers (lower row) after treatments with SF and/or CIS, or vehicle control, is shown at ×5 magnification and ×20 magnification in the insets; scale bars = 130 μm and 34 μm for the main photograph and the inset, respectively.

To assess whether SF might affect the sensitivity of HNSCC–CSC xenografts towards chemotherapy, we transplanted CD44^+^/CD271^+^ cells from SCC12 cell line into the tongue of nude immunocompromised mice. Mice were injected I.P with the vehicle (normal saline), SF, CIS or SF + CIS, and tumour growth was measured weekly for 49 days (Fig. 5a). When compared with the control group, treatment with either SF or CIS alone inhibited tumour growth and tumour volumes by 59% or 54.5%, respectively (Fig. 5b). SF + CIS treatment reduced tumour volume by 73% (Fig. 5b). A significant difference in tumour size was observed between mice in the control group and those in the treatment groups starting from 14 days after treatment. There was a significant difference between the SF + CIS treatment group and the SF or the CIS group after 35 days (Fig. 5b). SF had no toxic effects on mice injected with either SF alone or combined with CIS, as shown by their body weights when compared with the sham-treated mouse group. There was an insignificant decrease in the body weights of mice receiving the combined treatment during the administration period; however, these mice re-gained their weights after treatment cessation. Mice in the control (saline-injected) group showed a marked reduction in their body weights during the follow-up (Fig. 5c). Histological analysis showed no tissue necrosis of the livers and kidneys in all groups of mice (Fig. 5d).

## Discussion

### Therapeutic efficacy of SF

In a previous study, we demonstrate that SF increased chemotherapeutic cytotoxicity of CIS and 5-FU against HNSCC.^13^ Our results were in line with other studies on oral cancers^18,19^ and in a variety of other types of cancers.^20,21^ However, little is known on the effect of combining SF on HNSCC–CSCs. In our previous study, we suggested that both CD44^+^ and CD271^+^ were suitable markers to isolate CSCs from HNSCC.^16^ In the current study, we used these FACS-sorted CD44^+^/CD271^+^ CSCs to examine the effect of SF/chemotherapy-combination treatments. Our results demonstrated that SF had a cytotoxic effect on HNSCC–CSCs that were elevated in both dose- and time-dependent manners. Other studies in oral carcinomas^22^ and other cancer types^23–25^ reported comparable results. The new finding of this study was that SF could be used as a combination treatment to enhance the toxicity of CIS and 5-FU against the more resistant CSCs in HNSCC. Adding 3.50 µM of SF nearly doubled the effect of CIS and multiplied the effect of 5-FU by 10 times, especially at lower chemotherapy doses. A concentration of 3.50 µM SF in the human body can be achieved simply by eating fresh broccoli sprouts. It was reported that following the ingestion of 40 g of broccoli, the SF plasma concentration reached 2.50 µM/L within 3 h.^26^ Remarkably, SF cytotoxic effect was comparable on both cell lines tested in this study (SCC12 and SCC38), even if SCC38 was known as a more chemoresistant cell line.^27,28^ This suggested that SF could affect CSCs from both chemoresistant and chemosensitive HNSCC.

Our results demonstrated that 3.5 µM of SF alone reduced CSC clonogenicity to the same extent as 0.5 µg/ml CIS, and was more efficient than 1.3 µg/ml of 5-FU. Furthermore, combining SF to the standard CIS or 5-FU chemotherapy treatments eliminated CSC clonogenic ability completely. Similar results were reported with Gemcitabine in pancreatic cancer, Taxol on prostate cancer^21^ and CIS on gastric cancer.^20^ We obtained comparable results with the sphere- formation assay. The dose of 3.5 µM tested was comparable to the range of 0.5–10 µM that had been used in other types of cancer to inhibit tumour-sphere formation.^21,24,25^

By using the annexin V/7-AAD assay, we found that SF treatment significantly increased early apoptosis in treated CSCs, which was equal to using 0.5 µg/ml CIS and greater than 1.3 µg/ml 5-FU. However, the combined treatment of SF and low doses of CIS or 5-FU led to increased apoptosis as compared with using a single chemotherapeutic drug or SF as a treatment. These results suggested that SF acted through multiple mechanisms to target CSCs, and that strategy could reduce the chance for CSCs to develop resistance against SF. SF induction of apoptosis on CSCs was also reported with pancreas and prostate CSCs.^21,23^

Our results demonstrated that the SF + CIS combination reduced tumour size that was formed by the inoculation of HNSCC–CSCs in the tongue of immunocompromised mice, as compared with mice treated with SF or CIS alone. All tumour-bearing mice had decreased body weights when compared with the sham-treated group, and they were highly significant with the control group (treated with saline only). This could be explained by an increase in tumour size, which interfered with normal feeding habits, even with the use of a soft-food diet. SF biosafety was shown by H&E staining of the mice livers and kidneys, as there was no necrosis with SF alone or combined with CIS. Several studies reported similar biosafety profiles for SF combined with other drugs.^21,29,30^ To our knowledge, we are the first to show that SF enhanced the cytotoxicity of CIS and 5-FU towards HNSCC–CSCs.

### Safety of SF on non-cancerous human stem cells

Several studies demonstrated that SF had little-to-no toxicity on non-cancerous human (adult) cells.^13,22,31^ In the current study, we additionally assessed SF effect on human stem cells. We demonstrated that a concentration of 3.5 µM SF and less, either used alone or combined with CIS or 5-FU, did not affect the viability of human stem cells. Also, a concentration of 3.5 µM SF did not affect the multipotential differentiation capacity of human periodontal and dental pulp stem cells. Several studies had equally reported that low doses of SF did not affect the viability of mesenchymal stem cells and protected them from carcinogens.^32–34^

### Molecular mechanism of SF-mediated targeting of HNSCC–CSCs

Mechanistically, we recently demonstrated that SF enhanced the cytotoxicity of chemotherapy (CIS or 5-FU) against HNSCC by stimulation of the caspase-dependent apoptosis pathway.^13^ In the current study, we reported comparable results on HNSCC–CSCs, such as SF increased the apoptotic effect of CIS and 5-FU on CSCs by inhibiting BCL2. Also, we found that SF increased the expression and activation of Caspase 3, both at the genomic and protein levels. Numerous other molecular mechanisms were suggested for the pro-apoptotic effect of SF, such as the cleavage of caspase-8 in pancreatic cancer,^35^ the fragmentation of the DNA-repairing protein poly (ADP-ribose) polymerase (PARP) and decreased expression of BCL2 in mammary, prostate and colon cancers.^36–38^

Aldehyde dehydrogenase 1 (ALDH1) is a member of the aldehyde dehydrogenase family of cytosolic isoenzymes, which are highly expressed in many types of stem and progenitor cells.^39^ Interestingly, ALDH1^+^ HNSCC cells showed a high self-renewal ability along with increased tumour formation, invasion and treatment resistance.^40^ It was reported that ALDH1 stimulated tumour proliferation and survival by activating Akt and c-MYC through the regulation of retinoic acid formation.^41,42^ The inhibition of tumour proliferation in our study might be explained partially by the reduction in ALDH1A1 gene expression. It was suggested that the dysregulation of self-renewal pathways in CSCs, such as SMO, NOTCH1 and BMI1, could be the cause for CSC tumorigenicity and treatment resistance.^43–45^ Studies have reported that chemotherapies using CIS and 5-FU might cause the selection of CSCs and increased the expression of self-renewal and drug resistance-related genes, like BMI1^46,47^ or ALDH1A1,^21,48,49^ which were also found in our study. Our in vitro experiments demonstrated that SF treatments prevented CIS and 5-FU to induce BMI1 and ALDH1A1 expression, and enhanced the downregulation of SMO, GLI1 and NOTCH1. Therefore, SF co-treatments contributed to the resensitisation of CSCs to chemotherapeutic drugs. Interestingly, a similar effect was reported in other cancer types, either with gemcitabine or cisplatin.^20,21^

The octamer-binding transcription factor 4 (OCT4) was suggested to be the best indicator for stemness and maintenance of an undifferentiated state.^50^ In a recent meta-analysis study, a strong correlation was found between OCT4 overexpression and poor overall survival of HNSCC patients.^51^ SOX2 overexpression was also reported to affect the invasion and metastasis induction of laryngeal squamous cell carcinomas.^52^ Our results showed that SF inhibited the expression of both SOX2 and OCT4.

In conclusion, we demonstrated that SF strongly enhanced the cytotoxic effect of the chemotherapeutic agents CIS and 5-FU against HNSCC–CSCs. Combining SF with either CIS or 5-FU also decreased the expression of self-renewal and drug resistance-related genes. Our data suggest that SF enhanced the effect mediated by chemotherapy, both in vitro and in vivo, and thus allowed a lowered dose of these chemotherapeutic agents. Combining SF to standard chemotherapy (CIS and 5-FU) may provide a better treatment modality option for the clinical setting.

## Supplementary information

Broccoli Extract Increases Drug-mediated Cytotoxicity Toward Cancer Stem Cells of Head and Neck Squamous Cell Carcinoma

## Data Availability

All data generated or analysed during this study are included in this published article.
