# Supplementary material for: Broccoli extract increases drug-mediated cytotoxicity towards cancer stem cells of head and neck squamous cell carcinoma
Source: Br J Cancer. 2020 Aug 10;123(9):1395–403. doi: 10.1038/s41416-020-1025-1 (PMC7591858; doi:10.1038/s41416-020-1025-1)
Supplement: Supplementary file 1 — Broccoli Extract Increases Drug-mediated Cytotoxicity Toward Cancer Stem Cells of Head and Neck Squamous Cell Carcinoma [file 41416_2020_1025_MOESM1_ESM.docx]

**Broccoli Extract Increases Drug-mediated Cytotoxicity Toward Cancer Stem Cells of Head and Neck Squamous Cell Carcinoma**

Osama Elkashty, Simon D. Tran

**Appendix:**

**Fluorescence‑activated cell sorting (FACS)**

Tumor cells were harvested using Accutase™ Cell Detachment Solution (BD Bioscience) and resuspended as a single-cell suspension in staining buffer (1% FBS in ice-cold PBS) with a final concentration of 1x10^6^ cells/100 µl. Cells were then incubated with the Fixable Viability Stain 450 (BD Bioscience) for 15 min on ice, protected from light and washed twice. Cells were blocked by incubation with Human TruStain FcX™ (Biolegend) for 10 min, then washed once. Cells were incubated with the antibodies for CD44 and CD271 at a dilution of 1:20 for 30 min on ice, protected from light, then washed twice.

**Real-Time qRT-PCR**

The first-strand cDNA was synthesized from 1 μg total RNA using High-Capacity cDNA Reverse Transcription kit (Thermo Fisher). For the quantification of gene amplification, qRT-PCR was performed using StepOnePlus™ Real-Time PCR System (Thermo Fisher) in the presence of PowerUp SYBR Green Master Mix (Thermo Fisher). The following gene-specific primers were used:

GAPDH: (5’-GAGAAGGCTGGGGCTCATTT-3’, 5’-AGTGATGGCATGGACTGTGG-3’), BMI-1: (5’-TCCTTAACAGTCTCAGGTATCAACC-3’, 5’-CACAGTTTCCTCACATTTCCA-3’), SMO: (5’-TGGTCACTCCCCTTTGTCCTCAC-3’, 5’-GCACGGTATCGGTAGTTCTTGTAGC-3’), GLI1: (5’-TTGGAGAAGCCGAGCCGAGTATC-3’, 5’-GAGTAGACAGAGGTTGGGAGGTAAGG-3’), NOTCH1: (5’-GCAGAGGCGTGGCAGACTAT-3’, 5’-ACTTGTACTCCGTCAGCGTG-3’), SOX2: (5’-ACACCAATCCCATCCACACT-3’, 5’-CAAACTTCCTGCAAAGCTCC-3’) OCT4: (5’-CTCGAGAAGGATGTGGTCCG-3’, 5’-GAAGTGAGGGCTCCCATAGC-3’), ALDH1A1: (5’-ATCAAAGAAGCTGCCGGGAA-3’, 5’- GCATTGTCCAAGTCGGCATC-3’), BCL2: (5’-CTGCACCTGACGCCCTTCACC-3’, 5’-CACATGACCCCACCGAACTCAAAGA-3’), BAX: (5’-CGGGTTGTCGCCCTTTTCTA-3’, 5’-TGGTTCTGATCAGTTCCGGC-3’) and Caspase3: (5’-CTCGGTCTGGTACAGATGTCGA-3’, 5’-CATGGCTCAGAAGCACACAAAC-3’).
